# Personal

**Published:** 1904-02

**Authors:** 


### PERSONAL.

---

Dr. William House, of Weston, Oregon, formerly of Buffalo, has accepted an appointment as associate medical director of Mount Tabor Sanitarium at Portland, Oregon.

---

Dr. Floyd S. Crego, of Buffalo, gave a dinner to Brigadier-General L. W. Pettibone and other members of his staff at the Buffalo Club, Wednesday evening, January 13, 1904. Other guests were General Samuel M. Welch, Colonel of the 65th, and Drs. Roswell Park, Charles G. Stockton, and A. H. Briggs. Dr. Crego retires from the position of surgeon (Lieut.-Colonel) of the Fourth Brigade, N. G. N. Y., which he has held for several years.

---

Dr. William C. Krauss, of Buffalo, was elected president of the Medical Society of the County of Erie during its annual meeting held January 12, 1904.

---

Dr. Willis G. Gregory, of Buffalo, was elected president of the State Board of Pharmacy during its last annual meeting held at Albany, January 4, 1904.

---

Dr. Charles L. Vaux, of Buffalo, who graduated in medicine here in 1902, has been appointed junior physician at the Manhattan State Hospital and left for his post of duty at New York, early in January. Dr. Vaux served a term as interne at the Buffalo Hospital of the Sisters of Charity, during which time after examination his name was placed upon the state civil service eligible list, from which the appointment above referred to was recently made.

---

Dr. Thomas Darlington, of New York, a practitioner of standing and a physician of skill, has been appointed president of the New York City Board of Health by the new Tammany administration.

---

Dr. John McGaw Woodbury has been reappointed commissioner of street cleaning by Mayor McClellan. Dr. Woodbury has proved himself one of the most efficient heads the New York city street cleaning department ever had.

---

Dr. Chauncey Pelton Smith, of Buffalo, formerly surgeon to the Lackawanna Steel Company, has resumed the practice of his profession at 481 Franklin street. Telephone, Tupper 412.

---

Dr. James F. W. Ross, of Toronto, is spending the winter in Egypt. He expects to return to his home about April 1.

---

E. G. Swift, of Detroit, has been appointed general manager of the great house of Parke, Davis & Company, vice William M. Warren, deceased. Mr. Swift brings matured experience to his new and responsible position derived from long service as a subordinate of Mr. Warren. We feel sure that the business will progress under Mr. Swift's administration.

---

Dr. Charles A. L. Reed, of Cincinnati, was the guest of honor at the annual meeting of the Columbus (O.) Academy of Medicine, December 21, 1903, at which he delivered an address entitled, Doctors in politics. The attendance was large and Dr. Reed received the plaudits of the assemblage for his interesting, witty, and eloquent address.

---

Dr. Henry D. Ingraham, of Buffalo, was elected president of the Medical Union at its annual meeting, held in December, 1903. Dr. Ingraham has been ill of pneumonia during the past few weeks, but is nearly or quite well again.

Professor Johan von Mikulicz-Radecki, lecturing not long ago at Breslau on his recent trip to the United States, said that he found more fruitful ideas among the American surgeons than among the French and English. He added: "The time is past when we were the givers and Americans the receivers. The American character has as a fundamental feature unlimited self-confidence, and the American believes he can do anything he wishes."

Professor Mikulicz's visit to Buffalo last April, at which time a dinner was tendered him, will be remembered pleasantly by everyone who was privileged to meet him.

---

Dr. William C. Krauss, of Buffalo, has been elected president of the professional staff of the Emergency Hospital at the recent annual meeting of the physicians who serve that institution.

---

Dr. Eugene A. Smith, of Buffalo, has been appointed surgeon of the fourth brigade, N. Y. N. G., with the rank of lieutenant colonel, vice Lieutenant Colonel Floyd S. Crego, M. D., retired at his own request. Dr. Smith's service in the Spanish war as a field officer will add to his other qualifications as chief medical officer of the command to which he has been appointed.

